# How surgical care is changing in the technological era

**DOI:** 10.4155/fsoa-2016-0010

**Published:** 2016-03-16

**Authors:** Marcello Migliore

**Affiliations:** 1 Thoracic Surgery, Department of General Surgery & Medical Specialties, University of Catania, Catania, Italy

**Keywords:** education, extended operation, medical technology, research, smartphones, tablet, uniportal VATS

It is undoubted that in the last 20 years technology has changed surgical care, which is now evolving rapidly following the introduction and expansion of minimally invasive surgery and the utilization of tablets and smartphones to communicate between patients and doctors, or between doctors themselves (mobile healthcare). In this article, I try briefly to analyze how these two major changes may bring a new dimension to the future of worldwide healthcare, using the example of thoracic surgery.

To begin, the introduction in 1998 of the ‘uniportal’ surgery today permits us to perform many operations in thoracic and abdominal surgery through a single small skin incision [1–6]. Uniportal video-assisted surgery is more and more often used worldwide, and is currently performed by a team formed by one or two surgeons. This is less than 20 years ago, and therefore it can be envisioned that in the future we could certainly need fewer surgeons [7]. Moreover, the fact that surgical skills needed to operate through a small incision must be prominent, it will be necessary for the surgeon to undergo specific training to acquire the necessary elevated surgical capability to operate on patients [2]. I envisage, for the benefit of our patients, that every complex operation could be watched by ‘expert surgeons’ who, in a supervisory room, act as live councilors for when an intraoperative doubt arises – this could decrease intraoperative risks and give better long-term results. Of note here is that use of smaller incisions to treat cancer should never place patients at risk, and the uniportal surgery would require approval by scientific societies following evidence of improvement of patient outcomes, or at least maintenance of the same outcomes, for benign and malignant diseases. I agree with the unpublished words of T Treasure that these techniques are not for every surgeon to explore, but if proven to be in a patient's best interests, they should not be avoided but adopted. The big question is therefore how to test whether innovations are true improvements, using randomized trials. Another point of future discussion is how to train our next generation of surgeons, who must use both hands to operate through a small entry in the chest or abdomen; of course, this will be facilitated in the case of robotic surgery as the surgeon will operate via the console.

Next, we will consider the use of mobile technology such as smartphones or tablets in medicine, which permit that images files (x-rays, CT-scan, angiography etc.) could be transferred through social media applications such as Whatsapp; the consultant can therefore see the images within a few seconds from home or wherever he/she is, and consequently medical opinions can be delivered to the resident without hesitation and without the need to reach the hospital, as was required 20 years ago [8–11]. It is therefore clear that the surgeon on-call with his own tablet does not need to travel in to hospital to see x-rays and so on. It could be possible that in the future patient conditions in the ward could be monitored from home via a camera, and it will be possible to talk with patients from home in order to monitor them, including nonconscious signals from the patient. The ‘on calls’ will certainly be less stressful if the doctor does not need to travel in the night to speak with the patient or to see x-rays and so on, and family life will be easier. I predict that the use of tablets and smartphones in surgical care will become standard practice because of the multifactorial evident advantages for patients, residents, consultants, hospital management and family life [10]; the value of instant messaging service applications to simultaneously inform all components of the team regarding the clinical situation and decisions taken about every single patient in the unit; and the positive extra care obtainable, also through specialized apps, to remote patients who lives in more rural areas where there is not, for example, a thoracic unit [10,11]. Nevertheless, there is still no well-documented evidence to support that the use of smartphones or tablets in the clinical practice could give true advantage to our patients.

Although technology has an important role in the advancement of healthcare, research is essential as it serves to demonstrate that surgical innovations given by technology are ‘true’ advantages for patients and surgeons. Worldwide collaboration will permit us to collect more patients and store clinical information in large databases, which could serve for translational research purposes. There is a tentative feeling that in the future indications for surgery based on ‘personal experience’ will count less and less as prospective randomized trials will help us to find definite answers for seemingly never endingly disputed topics, such as surgery for mesothelioma or lung metastases [12–14]. Regarding this matter, Treasure *et al*. recently wrote that the E in EBM stands for Evidence, not for eminence, experience, expertise, eloquence or any words that have been used to give authority to one or a group of surgeons [15]. Extended operations such as extrapleural pneumonectomy for mesothelioma could be definitively stopped in favor of less-extensive surgeries, and questions regarding surgery for metastases for colorectal cancer will certainly have an answer after the PulMiCC trial ends.

For the above reasons, the next decades will witness more pioneering randomized trials that will serve to prove that the ‘theoretical’ advantages of uniportal surgery, extended oncologic surgeries and the use of mobile healthcare are ‘true’ advantages for patients and surgeons.
